# Is comprehensiveness critical? Comparing short and long format cognitive assessments in preclinical Alzheimer disease

**DOI:** 10.1186/s13195-021-00894-5

**Published:** 2021-09-13

**Authors:** Jason Hassenstab, Jessica Nicosia, Megan LaRose, Andrew J. Aschenbrenner, Brian A. Gordon, Tammie L. S. Benzinger, Chengjie Xiong, John C. Morris

**Affiliations:** 1 Charles F. and Joanne Knight Alzheimer Disease Research Center, Department of Neurology, Washington University School of Medicine, St. Louis, MO USA; 2 Department of Psychological & Brain Sciences, Washington University in St. Louis, St. Louis, MO USA; 3 Department of Radiology, Washington University School of Medicine, St. Louis, MO USA; 4 Division of Biostatistics, Washington University School of Medicine, St. Louis, MO USA

**Keywords:** Alzheimer disease, Cognitive decline, Cognitive assessment

## Abstract

**Background:**

Comprehensive testing of cognitive functioning is standard practice in studies of Alzheimer disease (AD). Short-form tests like the Montreal Cognitive Assessment (MoCA) use a “sampling” of measures, administering key items in a shortened format to efficiently assess cognition while reducing time requirements, participant burden, and administrative costs. We compared the MoCA to a commonly used long-form cognitive battery in predicting AD symptom onset and sensitivity to AD neuroimaging biomarkers.

**Methods:**

Survival, area under the receiver operating characteristic (ROC) curve (AUC), and multiple regression analyses compared the MoCA and long-form measures in predicting time to symptom onset in cognitively normal older adults (*n* = 6230) from the National Alzheimer’s Coordinating Center (NACC) cohort who had, on average, 2.3 ± 1.2 annual assessments. Multiple regression models in a separate sample (*n* = 416) from the Charles F. and Joanne Knight Alzheimer Disease Research Center (Knight ADRC) compared the sensitivity of the MoCA and long-form measures to neuroimaging biomarkers including amyloid PET, tau PET, and cortical thickness.

**Results:**

Hazard ratios suggested that both the MoCA and the long-form measures are similarly and modestly efficacious in predicting symptomatic conversion, although model comparison analyses indicated that the long-form measures slightly outperformed the MoCA (HRs > 1.57). AUC analyses indicated no difference between the measures in predicting conversion (DeLong’s test, *Z* = 1.48, *p* = 0.13). Sensitivity to AD neuroimaging biomarkers was similar for the two measures though there were only modest associations with tau PET (*r*s = − 0.13, *p*s < 0.02) and cortical thickness in cognitively normal participants (*r*s = 0.15–0.16, *p*s < 0.007).

**Conclusions:**

Both test formats showed weak associations with symptom onset, AUC analyses indicated low diagnostic accuracy, and biomarker correlations were modest in cognitively normal participants. Alternative assessment approaches are needed to improve how clinicians and researchers monitor cognitive changes and disease progression prior to symptom onset.

## Introduction

Detecting changes in cognition in the earliest stages of Alzheimer disease (AD) in longitudinal studies requires that assessment measures meet several criteria. Assessments must be psychometrically valid and reliable and test relevant domains of cognition such as episodic memory, executive function, attentional control, processing speed, language, and orientation. For both overall scores and domain-specific scores, these measures should have good retest reliability and inter-rater reliability. Of particular importance, especially for studies that enroll large asymptomatic cohorts, is that the tests included are sensitive enough to detect subtle changes in cognition that characterize the preclinical asymptomatic stages of AD [1, 2]. Attempting to maximize sensitivity, AD cohort studies and clinical trials typically involve extensive cognitive testing sessions including dozens of individual measures. The comprehensiveness of a cognitive evaluation is often valued as a method to provide more nuanced and sensitive insights into cognitive changes commonly associated with AD pathology [3, 4]. However, this comprehensiveness comes at a cost as lengthier evaluations can be problematic for multiple reasons. First, longer assessments increase participant burden and require significant personnel time for test administration, scoring, quality control, and data entry^1^. Second, day-to-day variability in stress and mood can significantly impact participants’ attention and fatigue, factors which have been shown to particularly influence older adults’ cognitive performance [5]. Finally, the tests which tap into the domains of cognition that decline in the earliest stages of AD, including episodic memory, attention, and working memory, are perceived as most taxing and effortful [6, 7] and often have suboptimal reliability [8].

Short-form cognitive assessment tools, sometimes referred to as cognitive screening measures, have been used for decades in clinical studies to provide a snapshot of overall cognitive and functional abilities and require a fraction of the time it takes to complete their long-form counterparts. The brevity and simplicity of these assessments make them popular tools among researchers and clinicians when assessing cognitive decline. The most common short-form measure in the AD literature is the Mini-Mental State Examination (MMSE) [9]. The MMSE requires approximately 10 min to administer and has high sensitivity and specificity for detecting symptomatic AD [10]. However, at milder stages of dementia, this test has exhibited poor sensitivity [11]. Specifically, due to ceiling effects, individuals with preclinical AD or early symptomatic AD (e.g., mild cognitive impairment [MCI] due to AD) are more likely to score within the “normal” range [12–14]. Beyond these validity and reliability issues, the MMSE is subject to copyright restrictions and carries fees for its use [15, 16], making it costly for large-scale studies and restricts research to investigators with sufficient resources.

For these reasons, the MMSE was not included in the National Alzheimer’s Coordinating Center (NACC) Uniform Data Set (UDS) in its third iteration (UDS 3) [17, 18] in lieu of the Montreal Cognitive Assessment (MoCA) [19], a short-form cognitive assessment similar to the MMSE with several advantages. The MoCA requires approximately 10–15 min to administer and tests seven domains of cognition: memory, visuospatial function, naming, attention, language, orientation, and abstraction. The most unique feature of the MoCA is that, apart from simple assessments of orientation and abstraction like those included in the MMSE, it uses items and test concepts drawn from classic long-form neuropsychological test batteries. The MoCA includes condensed versions, or a “sampling,” of more comprehensive measures including the trail making test, letter fluency, confrontation naming, digit span, and verbal list learning and recall. Like the MMSE, it is highly sensitive to dementia, but unlike the MMSE, it is more sensitive to early symptomatic AD [14, 20]; has shown sensitivity to AD biomarkers [21]; and is available at no cost for non-profit use, although its publisher has recently begun requiring training and certification fees (https://www.mocatest.org/training-certification/).

The goal of the current study was to determine how the short-form MoCA measure compares to standard long-form neuropsychological tests in terms of predicting the onset of symptomatic dementia in the NACC cohort. The NACC cohort is a large, well-characterized sample of older adults enrolled in ongoing studies of aging and dementia at ~ 30 Alzheimer's Disease Research Centers across the USA. Additional analyses were done using participants enrolled in studies at the Charles F. and Joanne Knight Alzheimer Disease Research Center (Knight ADRC) to determine the sensitivity of the short-form and long-form measures to AD neuroimaging biomarkers, including amyloid positron emission tomography (PET), tau PET, and magnetic resonance imaging (MRI) structural measures. We hypothesized that, in comparison with the MoCA, the long-form cognitive measures from the UDS 3 would show superior specificity and sensitivity in predicting conversion to symptomatic AD and would be more sensitive to AD neuroimaging biomarkers.

## Study 1

### Methods

#### Participants

The NACC UDS 3 is a standardized evaluation consisting of clinical and cognitive measures administered to participants enrolled in ongoing studies of aging and dementia at ~ 30 centers funded by the National Institute on Aging (NIA) Alzheimers Disease Research Center (ADRC) program. Written informed consent is obtained at the individual ADRCs and approved by individual Institutional Review Boards (IRBs). We included participant visits submitted to NACC by the ADRCs from March of 2015 to August 2019. This time period reflects when the MoCA and other UDS 3 measures were introduced into the ADRCs and submitted to NACC. Because we were interested in determining the utility of the MoCA as compared to the long-form UDS 3 cognitive battery in predicting disease progression from cognitive normality to onset of symptomatic disease, we included only participants who were cognitively normal at their first visit when MoCA was introduced into the ADRCs. Participants were required to have a Clinical Dementia Rating™ (CDR™) [22] of 0 at their first visit and have at least one follow-up visit.

#### Clinical and cognitive measures

Clinical status was determined with the CDR which uses a 5-point scale to characterize six domains of cognitive and functional performance that are applicable to AD and other dementias [22]. The domains include memory, orientation, judgment and problem solving, community affairs, home and hobbies, and personal care. CDR scores are determined through semi-structured interviews with the participant and a reliable informant such as a family member or friend. A CDR score of 0 indicates cognitive normality, 0.5 = very mild dementia, 1 = mild dementia, 2 = moderate dementia, and 3 = severe dementia.

The UDS 3 cognitive battery includes measures of episodic memory (Craft Story 21, Benson Complex Figure Recall), language (the Multilingual Naming Test (MINT)), visuospatial functioning (Benson Complex Figure Copy), immediate attention (Trails A, Number Span Forward), working memory (Number Span Backwards), and executive functioning (Trails B; see Weintraub et al., 2018 for a detailed description and associated references for individual measures). Each test was standardized using the mean and standard deviation from the first visit for individuals who remained CDR 0 (i.e., non-converters) to form *Z*-scores. *Z*-scores from each test were then averaged together to form domain scores (i.e., memory, visual, attention, language) which were then averaged together to form a simple global composite score. Because the MoCA total score is commonly used in the diagnosis of cognitive impairment [23], our analyses focused on comparing the MoCA total score with the UDS 3 global composite score.

#### Statistical analyses

Time to symptomatic conversion was operationalized as the time, in years, from participants’ initial study time point to the time point in which they were first determined to have a non-zero CDR score (or, in the case of individuals who never converted to a non-zero CDR, their final time point in the study). Individuals who died during follow-up were not included in the analyses (i.e., 148 non-converters and 31 converters). Two cut points, one at − 1 SD and one at − 1.5 SDs, were used for both the MoCA (scores of 23 and 21, respectively) and the UDS 3 global composite score. Similar cut points have been used in the literature to indicate the presence or absence of cognitive impairment [24–28]. Log-rank tests were used to compare the Kaplan-Meier survival curves for high and low groups for each measure (i.e., the MoCA and the UDS 3 global composite). Adjusted hazard ratios (HRs) and their 95% confidence intervals were estimated using a Cox proportional hazards model with covariates including baseline age, self-reported gender, years of education, and presence of one or more apolipoprotein ε4 (APOE4) alleles with the *survival* package version 3.2 in the R statistical computing environment [29].

To obtain measures of the two tests’ diagnostic discrimination abilities [30], the scales were subjected to an area under the curve (AUC) analysis of receiver operating characteristics (ROC) curves using the *pROC* package version 1.17 in R [30]. This analysis focused on examining which score provided better diagnostic accuracy in predicting conversion status using baseline data from individuals who were CDR 0 at their initial visit. The ROC analysis results were interpreted following the diagnostic accuracy guidelines from Swets (1996) such that an AUC < 0.70 indicates low diagnostic accuracy, an AUC in the range of 0.70–0.90 indicates moderate diagnostic accuracy, and an AUC ≥ 0.90 indicates high diagnostic accuracy [30]. DeLong’s test for correlated ROC curves was used to test whether the two areas under the curve were significantly different from one another.

Finally, multiple regression model comparison analyses were used to directly compare the efficacy of the baseline, continuous MoCA, and UDS 3 global composite scores in predicting the time to symptomatic conversion. Because the MoCA and UDS 3 global composite were correlated with one another, *r* = 0.70, *p* < 0.001, a single multiple regression model including both variables would be subject to potential multicollinearity issues. Therefore, non-nested models were used, and model fit was evaluated based on the *R*^2^, Akaike information criterion (AIC), Bayesian information criterion (BIC), and deviance values. For all models, males served as the reference group for the gender variable, and APOE negative served as the reference groups for the APOE variable.

### Results

#### Demographics

Six thousand two hundred thirty cognitively normal older adults aged 72.9 ± 10.4 years from the NACC cohort were followed for an average of 2.3 ± 1.2 annual assessments (range = 1–5 assessments). As expected, converters had more assessments than non-converters, *t*(746.92) = 12.90, *p* < 0.001, *d* = 0.55. Participants that enroll as cognitively normal that are observed for longer periods are more likely to convert due to advancing age (see Table 1 for additional demographic information).

**Table 1:** Study 1 demographic data

Converter	No, *N* = 5622^1^	Yes, *N* = 608^1^	***p***-value^2^
**Age**	73 (7)	76 (8)	< 0.001
**Gender**			0.002
Female	3709 (66%)	362 (60%)	
Male	1913 (34%)	246 (40%)	
**Education (years)**	16.24 (2.81)	16.08 (3.09)	0.24
**APOE status**			0.4
Neg.	2608 (70%)	351 (68%)	
Pos.	1134 (30%)	167 (32%)	
**Race**			0.21
White	4499 (80%)	480 (79%)	
Black or African American	853 (15%)	107 (18%)	
Asian	148 (2.6%)	10 (1.6%)	
American Indian or Alaska Native	40 (0.7%)	7 (1.2%)	
Native Hawaiian or other Pacific Islander	6 (0.1%)	0 (0%)	
Others	32 (0.6%)	2 (0.3%)	
Unknown	44 (0.8%)	2 (0.3%)	
**Number of visits**	2.25 (1.2)	3.21 (0.94)	< 0.001
**MoCA**	26.1 (2.9)	24.6 (3.3)	< 0.001
**UDS 3 global composite**	0.05 (0.57)	− 0.25 (0.59)	< 0.001

^1^Mean (SD); *n* (%)

^2^Welch two-sample *t*-test; Pearson’s chi-squared test

#### Survival model results

**Fig. 1:**
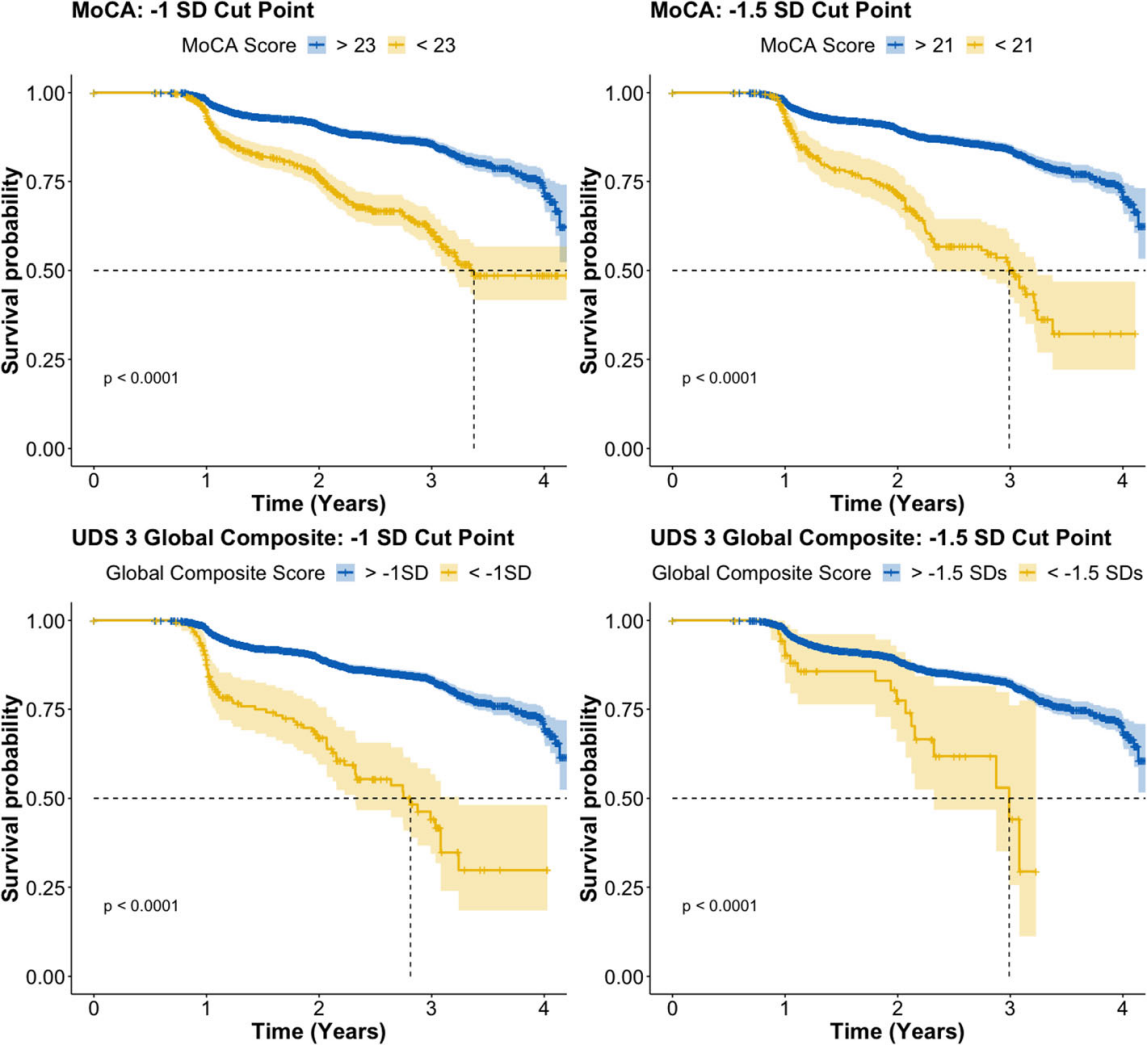
Survival curves for the MoCA and UDS 3 global composite for the two different cut points (− 1 SD and − 1.5 SDs)

Survival analyses were conducted using outcomes from the short-form MoCA and the long-form UDS 3 global composite to predict the time to symptomatic onset (defined as a change from CDR 0 to CDR > 0). Percentages of high- and low-scoring converters and non-converters for each cut point are presented in Table 2. Outcomes were contrasted on HR magnitude and their confidence intervals. For reference, higher HRs are interpreted as representing a better prediction of future conversion and narrower confidence intervals suggest less variability in prediction. Primary analyses contrasted individuals who scored above vs. below the − 1 SD and − 1.5 SDs cut points on the MoCA and UDS 3 global composite. HRs for symptomatic conversion by the cut point criteria described above, adjusted for demographic covariates, are presented in Table 3. For all models, the adjusted cumulative incidence of time to symptomatic conversion was increased in the individuals who scored below the cut point after controlling for demographic covariates as compared to individuals who scored above the cut point, HRs > 1.57, *p*s < 0.001. Comparison of HRs indicated that the UDS 3 global composite was slightly better at predicting symptomatic conversion than the MoCA with the − 1 SD cut point. However, the opposite was true (MoCA HR > UDS 3 global composite HR) with the − 1.5 SD cut point (see Table 3). Overall, these results ultimately suggest that both the MoCA and the UDS 3 global composite show relatively equivalent HRs and are thus similarly, yet modestly, efficacious in predicting symptomatic conversion. Kaplan-Meier curves for the MoCA and UDS 3 global composite at the two different cut points are presented in Fig. 1.

**Table 2:** Study 1 cut point *N*s

Converter	No, *N* = 5622^1^	Yes, *N* = 608^1^
**MoCA − 1 SD cut point**		
High	4710 (84%)	416 (68%)
Low	912 (16%)	192 (32%)
**Global composite − 1 SD cut point**		
High	5381 (96%)	543 (89%)
Low	241 (4.3%)	65 (11%)
**MoCA − 1.5 SD cut point**		
High	5204 (93%)	505 (83%)
Low	418 (7.4%)	103 (17%)
**Global composite − 1.5 SD cut point**		
High	5537 (98%)	591 (97%)
Low	85 (1.5%)	17 (2.8%)

^1^*n* (%)

**Table 3:** HRs for time to symptomatic conversion by task score adjusted for covariates

Predictor	MoCA − 1 SD cut point			MoCA − 1.5 SD cut point			Composite − 1 SD cut point			Composite − 1.5 SD cut point		
	HR	95% CI	***p***	HR	95% CI	***p***	HR	95% CI	***p***	HR	95% CI	***p***
**Age**	1.02	1.01, 1.04	< 0.001	1.03	1.01, 1.04	< 0.001	1.03	1.02, 1.04	< 0.001	1.03	1.02, 1.04	< 0.001
**Gender**	0.76	0.64, 0.91	0.003	0.72	0.60, 0.86	< 0.001	0.74	0.62, 0.89	0.001	0.74	0.62, 0.88	< 0.001
**Education**	1.00	0.97, 1.03	> 0.9	1.00	0.97, 1.04	0.90	0.99	0.96, 1.02	0.40	0.97	0.94, 1.00	0.035
**APOE**	1.22	1.01, 1.47	0.04	1.20	0.99, 1.44	0.06	1.23	1.02, 1.49	0.03	1.22	1.01, 1.47	0.038
**MoCA**												
High	–	–	–	–	–	–						
Low	2.99	2.45, 3.65	< 0.001	3.87	3.00, 4.99	< 0.001						
**Global composite**												
High							–	–	–	–	–	
Low							4.06	2.95, 5.58	< 0.001	2.69	1.57, 4.61	< 0.001

*HR* hazard ratio, *CI* confidence interval

#### ROC results

ROC analyses in the NACC sample indicated an area under the curve (AUC) estimate for the MoCA of 0.64 and an AUC estimate for the UDS 3 global composite of 0.66. DeLong’s test for two correlated ROC curves was non-significant, *Z* = 1.48, *p* = 0.13, indicating that these measures demonstrate similarly low diagnostic accuracy in predicting conversion to symptomatic AD [31].

#### Model comparison results

The multiple regression models predicting the time to symptomatic conversion indicated that both the continuous MoCA and UDS 3 global composite scores significantly predicted time to symptomatic conversion after covarying out effects of age, self-reported gender, years of education, and APOE4 status. The results of the model comparison analyses suggested that the model including the UDS 3 global composite score outperformed the model including the MoCA score in predicting time to symptomatic conversion. This was indicated by the larger *R*^2^ and smaller AIC, BIC, and deviance values (see Table 4).

**Table 4:** Study 1 model comparisons

Predictor	MoCA model			Global composite model		
	Beta	95% CI	***p***	Beta	95% CI	***p***
**Age**	0.00	− 0.01, 0.01	0.60	0.00	− 0.01, 0.01	0.80
**Gender**	0.13	− 0.02, 0.28	0.09	0.11	− 0.04, 0.26	0.14
**Education**	− 0.03	− 0.06, 0.00	0.05	− 0.03	− 0.06, − 0.01	0.01
**APOE**	0.06	− 0.10, 0.22	0.40	0.06	− 0.09, 0.22	0.40
**Standardized MoCA**	0.11	0.04, 0.18	0.003			
**Global composite**				0.30	0.17, 0.44	< 0.001
*R*^2^	0.028			0.05		
AIC	1292			1280		
BIC	1322			1310		
Deviance	362			353		

*CI* confidence interval

## Study 2

### Methods

#### Participants

Participants were community-residing volunteers enrolled in longitudinal studies of memory and aging at the Knight ADRC at Washington University in St. Louis. We were interested in determining the associations between the MoCA and long-form measures with AD neuroimaging biomarkers, so we selected participants based on the availability of at least 1 amyloid PET scan, 1 tau PET scan, and a volumetric MRI that were collected within 1 year of each other. We also sought to determine the sensitivity of the MoCA and long-form measures in tracking disease progression from the asymptomatic to symptomatic stages; thus, data were analyzed in two groups: participants who were CDR 0 and participants who were CDR > 0. All participants signed a standard informed consent document, and all procedures were approved by the Institutional Review Board at Washington University in St. Louis.

#### Clinical and cognitive measures

Measures included the CDR (as described in study 1), MoCA, and UDS 3 (as described in study 1). The UDS 3 global composite was calculated in the same way as described in study 1.

#### Neuroimaging

Amyloid PET imaging was performed with either florbetapir (18F-AV-45) or Pittsburgh Compound B (PiB) and was acquired on a Biograph mMR (Siemens Medical Solutions, Malvern, PA). All florbetapir PET scans include data 50- to 70-min post-injection, and all PiB PET scans include data 30- to 60-min post-injection. All data were converted to standardized uptake value ratios (SUVRs) with the cerebellar cortex used as a reference region before then being converted to the Centiloid scale [32, 33]. PET data were processed with an in-house pipeline using regions of interest derived from FreeSurfer (https://github.com/ysu001/PUP) [34]. This approach corrects for the spillover signal from adjacent regions of interest and non-brain tissue on the basis of the scanner point spread function and the relative distance between regions. This partial volume correction approach accounts not only for spillover from different areas in the brain but also for spillover from the non-brain regions into the brain. Amyloid deposition was quantified with the average across the left and right lateral orbitofrontal, medial orbitofrontal, rostral middle frontal, superior frontal, superior temporal, middle temporal, and precuneus regions. Tau PET imaging used the tracer 18F-AV-1451 (flortaucipir) and was acquired on a Biograph 40 PET/CT scanner (Siemens Medical Solutions). Data from the 80- to 100-min post-injection window were converted to SUVRs using a cerebellar cortex reference and partial volume corrected. Deposition was summarized with the average of the bilateral entorhinal cortex, amygdala, inferior temporal lobe, and lateral occipital cortex [35]. MRI data were acquired on a Siemens Biograph mMR or Trio 3T scanner. T1-weighted images were acquired with a magnetization-prepared rapid acquisition gradient echo sequence acquired in the sagittal orientation with a repetition time of 2300 ms, an echo time of 2.95 ms, a flip angle of 9°, 176 slices, an in-plane resolution of 240 × 256, and a slice thickness of 1.2 mm. Images underwent volumetric segmentation with FreeSurfer 5.3 (freesurfer.net) to identify the regions of interest for further analysis [36, 37]. Cortical thickness values were obtained for each hemisphere for a limited number of regions of interest (ROIs) reflecting the brain atrophy patterns in AD [38]. Cortical thickness was calculated as the shortest distance between the cortical gray/white boundary to the gray/CSF boundary [39].

#### Statistical analyses

The relationships between the AD neuroimaging biomarkers (at baseline) and the MoCA/UDS3 global composite in CDR 0 and CDR 0.5 individuals were examined using multiple regression models in the R statistical computing environment. As in study 1, due to the high degree of correlation between the MoCA and UDS 3 global composite scores, non-nested model comparison metrics (i.e., *R*^2^, AIC, BIC, and deviance) were used in lieu of a single, nested multiple regression model to avoid potential confounding multicollinearity issues. Separate models were run predicting each biomarker and separately for each subset of the data (i.e., CDR 0 s, CDR 0.5 s) using demographic predictor variables as the covariates and either the MoCA or the UDS 3 global composite as the key independent variable. For all models, males served as the reference group for the gender variable, and APOE negative served as the reference group for the APOE variable.

### Results

#### Demographics

Participants included 416 mostly older adults aged 45 to 92 years old enrolled in ongoing studies at the Knight ADRC (see Table 5 for the complete demographic information) and who had completed at least one relevant biomarker scan and a clinical assessment. Most participants (*n* = 365) were cognitively normal with a CDR of 0.

**Table 5:** Study 2 demographic data

CDR	0, *N* = 365^1^	0.5, *N* = 51^1^	***p***^2^
**Age**	71 (6)	74 (6)	< 0.001
**Gender**			0.12
Female	211 (58%)	23 (45%)	
Male	154 (42%)	28 (55%)	
**Education (years)**	16 (2)	16 (3)	0.076
**APOE**			0.001
Neg.	245 (67%)	22 (43%)	
Pos.	119 (33%)	29 (57%)	
**Race**			0.83
White	320 (88%)	45 (88%)	
Black or African American	41 (11%)	5 (9.8%)	
Others (American Indian, Alaska Native, Native Hawaiian, Pacific Islander, or Asian)	4 (1.1%)	1 (2.0%)	
**MoCA**	26 (2)	22 (4)	< 0.001
**UDS 3 global composite**	0.22 (0.58)	− 0.62 (0.73)	< 0.001
**Amyloid PET (Centiloid)**	17 (24)	59 (40)	< 0.001
**Tau PET SUVR**	1.22 (0.19)	1.64 (0.56)	< 0.001
**AD ROI cortical thickness (mm)**	2.82 (0.14)	2.64 (0.21)	< 0.001

^1^Mean (SD); *n* (%)

^2^Welch two-sample *t*-test; Pearson’s chi-squared test

#### Regression results

**Fig. 2:**
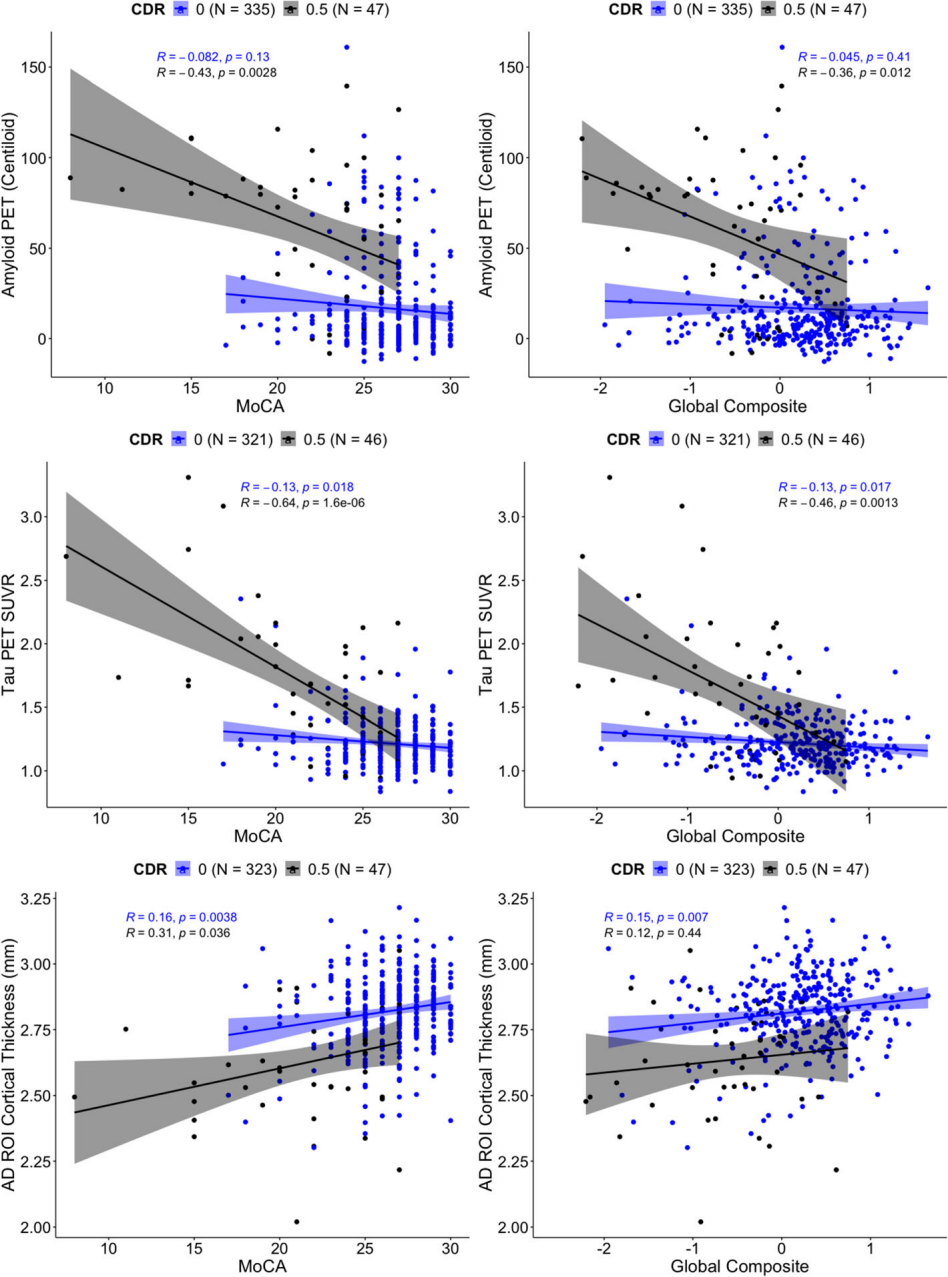
Biomarker correlations with MoCA/UDS 3 global composite by CDR group

As shown in Table 6, analyses of CDR 0 participants indicated small but significant relationships between both short- and long-form scores (i.e., the MoCA and UDS 3 global composite) and tau PET and cortical thickness (*p*s 0.007–0.03). Model comparison results indicated that the MoCA and UDS 3 global composite were relatively similarly sensitive in predicting biomarker values as indicated by the *R*^2^, AIC, BIC, and deviance values (see Table 6). As expected, in the CDR 0.5 subsample, there were stronger relationships between the MoCA and UDS 3 global composite and all of the neuroimaging biomarkers, including amyloid PET, as compared to the CDR 0 sample (see Table 7). Model comparison analyses indicated that the MoCA was a better predictor of biomarker levels as compared to the UDS 3 global composite in the CDR 0.5. Scatterplots showing the relationships between the MoCA and UDS 3 global composite score and the three neuroimaging biomarkers, with separate regression lines for CDR 0 and 0.5 s, are displayed in Fig. 2. As shown in the scatterplots, and as made evident by the regression models, both the MoCA and UDS 3 global composite were essentially equivalent in their associations with the neuroimaging AD biomarkers.

**Table 6:** Biomarker multiple regression models for CDR 0 s

Predictor	Amyloid PET (Centiloid)						Tau PET (SUVR)						AD ROI Cortical Thickness					
	Beta	95% CI	***p***	Beta	95% CI	***p***	Beta	95% CI	***p***	Beta	95% CI	***p***	Beta	95% CI	***p***	Beta	95% CI	***p***
**Age**	0.04	0.03, 0.06	< 0.001	0.04	0.03, 0.06	< 0.001	0.05	0.03, 0.06	< 0.001	0.05	0.03, 0.06	< 0.001	− 0.05	− 0.06, − 0.03	< 0.001	− 0.05	− 0.06, − 0.03	< 0.001
**Gender**	0.14	− 0.07, 0.35	0.20	0.12	− 0.09, 0.33	0.30	0.57	0.35, 0.78	< 0.001	0.52	0.31, 0.73	< 0.001	0.21	− 0.01, 0.42	0.056	0.24	0.03, 0.45	0.028
**Education**	0.02	− 0.03, 0.06	0.50	0.01	− 0.03, 0.06	0.60	0.04	− 0.01, 0.09	0.087	0.04	− 0.01, 0.08	0.12	− 0.06	− 0.11, − 0.01	0.013	− 0.06	− 0.10, − 0.01	0.013
**APOE**	0.56	0.34, 0.78	< 0.001	0.56	0.34, 0.78	< 0.001	0.32	0.10, 0.54	0.004	0.32	0.10, 0.54	0.005	− 0.10	− 0.32, 0.12	0.40	− 0.10	− 0.32, 0.12	0.40
**MoCA**	− 0.05	− 0.16, 0.06	0.30				− 0.15	− 0.26, − 0.04	0.007				0.13	0.02, 0.24	0.024			
**Global composite**				− 0.01	− 0.18, 0.17	> 0.9				− 0.2	− 0.38, − 0.02	0.028				0.22	0.03, 0.40	0.02
*R*^2^	0.128			0.126			0.165			0.158			0.133			0.134		
AIC	916			917			864			867			881			880		
BIC	942			943			891			893			907			907		
Deviance	291			292			267			269			278			278		

Imaging biomarkers standardized using CDR 0 means and SDs. Betas can be interpreted as the degree of biomarker change in SDs for every 1-unit change in the predictor

*CI* confidence interval

**Table 7:** Biomarker multiple regression models for CDR 0.5 s

Predictor	Amyloid PET (Centiloid)						Tau PET (SUVR)						AD ROI Cortical Thickness					
	Beta	95% CI	***p***	Beta	95% CI	***p***	Beta	95% CI	***p***	Beta	95% CI	***p***	Beta	95% CI	***p***	Beta	95% CI	***p***
**Age**	0.03	− 0.04, 0.10	0.40	0.03	− 0.04, 0.10	0.40	− 0.12	− 0.23, − 0.01	0.026	− 0.13	− 0.26, − 0.01	0.041	− 0.05	− 0.12, 0.01	0.11	− 0.05	− 0.12, 0.02	0.13
**Gender**	0.61	− 0.24, 1.5	0.20	0.63	− 0.24, 1.5	0.15	1.10	− 0.24, 2.3	0.11	1.10	− 0.44, 2.6	0.20	− 0.02	− 0.85, 0.81	> 0.9	− 0.05	− 0.94, 0.84	> 0.9
**Education**	0.02	− 0.18, 0.21	0.90	0.03	− 0.19, 0.25	0.80	0.22	− 0.08, 0.52	0.14	0.19	− 0.20, 0.58	0.30	− 0.18	− 0.37, 0.02	0.073	− 0.11	− 0.33, 0.12	0.40
**APOE**	1.2	0.37, 2.1	0.006	1.3	0.45, 2.2	0.004	0.85	− 0.48, 2.2	0.2	1.30	− 0.29, 2.8	0.11	− 0.04	− 0.90, 0.81	> 0.9	− 0.2	− 1.1, 0.70	0.70
**MoCA**	− 0.33	− 0.61, − 0.06	0.018				− 1.2	− 1.6, − 0.74	< 0.001				0.36	0.09, 0.63	0.009			
**Global composite**				− 0.85	− 1.7, − 0.04	0.041				− 2.4	− 3.8, − 1.0	0.002				0.45	− 0.39, 1.3	0.30
*R*^2^	0.383			0.362			0.572			0.406			0.206			0.088		
AIC	171			173			206			221			169			175		
BIC	184			185			218			233			182			188		
Deviance	77.6			80.2			173			241			74			85		

Imaging biomarkers standardized using CDR 0 means and SDs. Betas can be interpreted as the degree of biomarker change in SDs for every 1-unit change in the predictor

*CI* confidence interval

## General discussion

The present study compared the efficacy of a popular short-form cognitive measure (MoCA) against a global composite score created from the more comprehensive long-form UDS3 cognitive battery in predicting AD progression. AD progression was operationalized as the time to the onset of initial symptoms. Additionally, we compared the short- and long-form measures on their sensitivity to AD neuroimaging biomarkers. Using data from the NACC, we compared the survival analyses, ROC curves, and multiple regression models using the MoCA total score and a global cognitive composite to investigate whether one measure outperformed the other. In a sample of data from the Knight ADRC, which included several neuroimaging biomarkers in addition to both the MoCA and UDS 3 cognitive battery, we further investigated the predictive power of each score in predicting biomarker levels in participants who were cognitively normal (CDR 0) and in those with very mild dementia (CDR 0.5).

Our first hypothesis, that the long-form measure would outperform the MoCA in predicting disease progression, was supported, although both measures produced only modest associations. We compared commonly used standard deviation (SD) cut points, at − 1.0 SD and − 1.5 SD, for both measures. Similar cut points have been used in the literature to indicate the presence or absence of cognitive impairment [24–28]. HRs from the survival analyses indicated that both the MoCA and UDS 3 global composite were relatively equivalent in their sensitivity to symptomatic conversion, though ROC analyses indicated that neither reached the threshold for acceptable diagnostic accuracy. Model comparison analyses in the NACC sample indicated that the UDS 3 global composite outperformed the MoCA by only a slim margin.

It appears that longer, more comprehensive cognitive assessments may afford a marginal benefit over shorter cognitive screening measures like the MoCA when used to predict the time to symptomatic disease onset. In contrast, when stricter cut points were used (i.e., − 1.5 SDs), the MoCA outperformed the long-form composite score. These findings should be interpreted with caution, however, because there are far fewer cases at this low-performance threshold for the long-form global composite than the MoCA (see Table 2 and confidence interval magnitude in Fig. 1), which was expected given that the sample was selected based on a baseline CDR of 0. These data also corroborate existing studies which find that cutoff scores, which purportedly identify individuals at the greatest risk for cognitive decline and symptom onset [2], have unacceptably poor predictive power, even when coupled with AD biomarker information [40].

We also hypothesized that the long-form global composite would prove superior to the MoCA in associations with AD neuroimaging biomarkers including amyloid PET, tau PET, and an AD-specific ROI cortical thickness measure. However, analyses in the Knight ADRC sample did not support this hypothesis. There were only minor associations between the MoCA and long-form global composite measures and tau PET SUVR and cortical thickness in CDR 0 individuals. As expected, and consistent with prior work in this cohort [41, 42], more robust relationships were seen in CDR 0.5 s and significant effects extended to all three imaging markers, including amyloid PET. Although it may be somewhat surprising that amyloid PET was not significantly associated with either cognitive measure in the CDR 0 participants, this has been observed in previous studies. Specifically, relationships with amyloid and cognition are relatively inconsistent [43–45], and accumulating evidence suggests that changes in cognition in the preclinical stages of AD are predominantly driven by tau pathology and structural changes, rather than by amyloid alone [41, 46, 47].

### Limitations

The findings of this study should be considered in light of a number of limitations, which may be addressed in future studies. First, although it would be optimal to directly compare the performance of the short-form and long-form measures to one another in a single multiple regression, the high degree of correlation between the measures would result in potential multicollinearity issues if included in a single model together. Thus, our analyses were restricted to non-nested model comparison techniques. Second, we only compare baseline performance of the short-form and long-form measures here; it is possible that the long-form battery is more sensitive to decline than the short-form MoCA when assessing longitudinal change. Third, only a limited number of demographic covariates were included in the models presented. It is, of course, possible that the results may differ if other covariates were included. Finally, the participants included in the NACC cohort and the Knight ADRC cohort are composed of mostly highly motivated older adults who are comprehensively phenotyped and often engaged in imaging and fluid biomarker studies and therefore are not representative of the general population. Although a primary goal of the NIA ADRC programs and the Knight ADRC is to diversify enrollment to include underrepresented persons in aging and dementia research, the participants in these studies are primarily White and highly educated (see Tables 1 and 5).

### Conclusions

Ultimately, the MoCA and UDS 3 global composite exhibit relatively equivalent, but limited, sensitivity to symptomatic conversion. Thus, depending on the use case, choosing the MoCA over longer cognitive batteries may afford sufficient sensitivity while also reducing administration time, costs, and participant burden. On the other hand, longer and more comprehensive measures do provide some unique power in predicting disease progression, depending on the cutoff point used. A shortcoming of both approaches is that neither demonstrated acceptable classification accuracy nor robust biomarker relationships in cognitively normal participants. In order for researchers and clinicians to reliably detect AD pathology in preclinical individuals, novel assessment methodologies with increased sensitivity and reliability (such as ecological momentary assessment studies or paradigms which move away from the traditional “one-shot” approach [48]) are necessary.

## Data Availability

The datasets used and/or analyzed during the current study are available from the corresponding author upon reasonable request.

## Footnotes

1 Additionally, in light of the COVID-19 pandemic, it is worth noting that the length of long-form cognitive assessments may also make them less adaptable to remote testing.
